# A netting-based alternative to rigid sorting grids in the small-meshed Norway pout (*Trisopterus esmarkii*) trawl fishery

**DOI:** 10.1371/journal.pone.0246076

**Published:** 2021-01-28

**Authors:** Ole R. Eigaard, Bent Herrmann, Jordan P. Feekings, Ludvig A. Krag, Claus R. Sparrevohn

**Affiliations:** 1 National Institute of Aquatic Resources (DTU AQUA), Technical University of Denmark, Kgs. Lyngby, Denmark; 2 SINTEF Ocean, Trondheim, Norway; 3 UiT the Arctic University of Norway, Tromsø, Norway; 4 National Institute of Aquatic Resources (DTU AQUA), Technical University of Denmark, North Sea Science Park, Hirtshals, Denmark; 5 Danish Pelagic Producers Organisation, Copenhagen, Denmark; Technological Educational Institute of Western Greece, GREECE

## Abstract

A new bycatch reduction device, termed “Excluder”, is presented as an alternative to a traditional rigid sorting grid, mandatory in the small-meshed Norway Pout (*Trisopterus esmarkii*) trawl fishery in the North Sea. The fishery is a high-volume fishery with large vessels, large demersal trawls and catches up to 100 tons per haul of this small forage fish. The Excluder is a 30 m long netting-based sorting system, developed to reduce bycatch (70 mm square meshes) and improving on board gear-handling and safety. The Excluder was tested against a 5.8 m^2^ standard sorting grid (35 mm bar spacing) in a twin-trawl experiment from the commercial 70 m trawler “S364 Rockall”. Catch data were analysed by species and length using the catch comparison method. For all bycatch species analysed, the Excluder had significantly lower catches relative to the grid: herring (21%), whiting (6%), mackerel (5%), American plaice (70%), witch flounder (15%), and lesser silver smelt (71%). For Norway Pout there was a significant increase in the overall catch efficiency of 32%. These results are explained by a 10 cm smaller L50 (the length of fish with 50% probability of being rejected by the sorting system) of the Excluder and a 15 times larger sorting area, which reduces the risk of clogging and loss of function. With these documented effects of improved sorting and target species catch efficiency, implementation of the Excluder would improve sustainability and address two main barriers of the current Norway pout fishery that limit quota capitalization; a tendency for Norway pout to mix with herring and whiting and lowered catch rates from grid-clogging. Additionally, gear-handling and safety on board would be improved.

## 1. Introduction

### 1.1. Stock characteristics and challenges of resource exploitation

The annual total allowed catch (TAC) of Norway pout (*Trisopterus esmarkii*) from the greater North Sea commonly exceeds 200 000 tons [1]. However, this very large amount of forage fish is practically never caught, and for the most recent decade catches have on average only been about 30% of the TAC [1]. There are likely two main explanations behind this unusually low quota capitalization, which is considered a problem by management as well as by industry. Both explanations are related to the biology and behaviour of Norway pout, which is different from that of most other (fully) exploited forage fish in the North Sea. Pelagic forage fish such as sprat (*Sprattus sprattus*) and blue whiting (*Micromesistius poutassou*) naturally occur in large dense shoals and exhibit a strong herding reaction to the front part of trawl gears [2]. Consequently, these species can be fished with large herded volume (HD) trawl types [3], which enable very high catches per unit of effort (CPUE) and profitable fisheries even if fish price is low. In contrast, Norway pout is a small, short-lived Gadoid [4, 5], which tends to aggregate in smaller, patchy, near bottom schools that are dispersed across larger areas [6]. With this near-bottom and more evenly scattered distribution of stock biomass, Norway pout is generally fished with large herded area (HA) trawl types, where catches depend on towing time and swept area rather than on searching time [3, 7]. The resulting lower CPUEs and poorer profitability, when compared to most other forage fish, are likely a key explanation of the low quota capitalization in the Norway Pout fishery. Another behaviour related complication of resource exploitation is the tendency for Norway pout to mix with similar sized gadoids of other species, mainly juveniles of whiting (*Merlangius merlangus*), and with herring (*Clupea harengus*) [8–10]. This makes it difficult for a trawl fishery to avoid unwanted bycatch, and hence to fish sustainably and comply with fisheries management regulations, which is likely a second key explanation of the low quota capitalization.

### 1.2. Fishery, management and technical regulations

Despite the technical challenges related to its resource exploitation, Norway pout has been the target species of a small-meshed North Sea trawl fishery (typically with codend mesh sizes from 16–31 mm) since the 1970ies [11]. Denmark and Norway are the two dominating fishing nations, traditionally accounting for more than 98% of the total annual catches (Denmark ~70–80% and Norway ~20–30%) [11]. The main seasons are 3^rd^ and 4^th^ quarters of the year and the main fishing areas are Fladen Ground and the edge of the Norwegian Trench. Norway pout is landed for reduction purposes (fish meal and fish oil) and fishing is generally carried out with large vessels and trawls and trip durations of approx. a week, resulting in very large catch volumes. The individual trawl hauls typically span 8–10 hours with variable catch-volumes in the range from 20 to 50 tons, but occasionally as high as 100 tons. As a result of bycatch issues a sorting grid with a maximum of 40 mm bar distance has been mandatory in the Norwegian fishery since 2010 and in the Danish fishery a grid with a maximum of 35 mm has been mandatory since 2012 [11]. The grids used in the Danish fishery are to be made out of a low-flexible material such as steel or rigid plastic. A previous experiment with a 23mm grid for the Danish Norway pout fishery [12] demonstrated gadoid bycatch reductions between 80.9 and 100%, but also target species loss of up to 13.7%. The larger bar spacing of 35 mm currently in the legislation is assessed by Danish authorities to offer a better trade-off between release of unwanted bycatch and loss of target species (i.e. less target species loss, but also less bycatch reduction). The use of species and size sorting grids to reduce unwanted catches in commercial fisheries has been extensive [7, 13], but despite many examples with successful by-catch reduction, the use of solid material grids is often met with apprehension from fishermen due to operational and safety concerns [14, 15]. This is also the case for the Norway pout fishery where the sorting grids (steel or rigid plastic) are large due to the size of the trawls employed in the fishery. The recent introduction of twin trawls in the fishery, in an attempt to increase catch rates, has also increased safety concerns when having to handle, not only one, but two heavy grids in rigid material. Consequently, industry initiated attempts have been made to develop alternative selection devices, which could match the sorting features of the current grid and at the same time improve safety and gear-handling on board.

### 1.3. Experimental objectives

The Excluder represents an innovative industry-driven development of a grid alternative, which has been designed by Greenline Fishing Gear. The Excluder is essentially a 30 meter lined tube characterized by being flexible, and made only of netting and PVC without any rigid materials. The design strategy behind the Excluder is to ensure that it can easily be reeled on the net drums and generally be handled without safety concerns. Furthermore, since the Excluder is designed with a considerably larger selection area than that of rigid grids, it is hypothesized that while improving safety and handling, the Excluder will also improve the size-based separation of target and bycatch species compared to a grid sorting system (i.e. enable a more optimal trade-off between maximizing release of unwanted bycatch and minimizing loss of target species). Here we describe the testing of these hypotheses in a twin-trawl experiment (the Excluder against a standard 35 mm rigid grid as used in the Danish fishery), conducted from the commercial 70 m trawler “S364 Rockall” in the North Sea in November 2018.

## 2. Materials and methods

### 2.1. Fishing vessel, fishing grounds and catch

**Fig 1:**
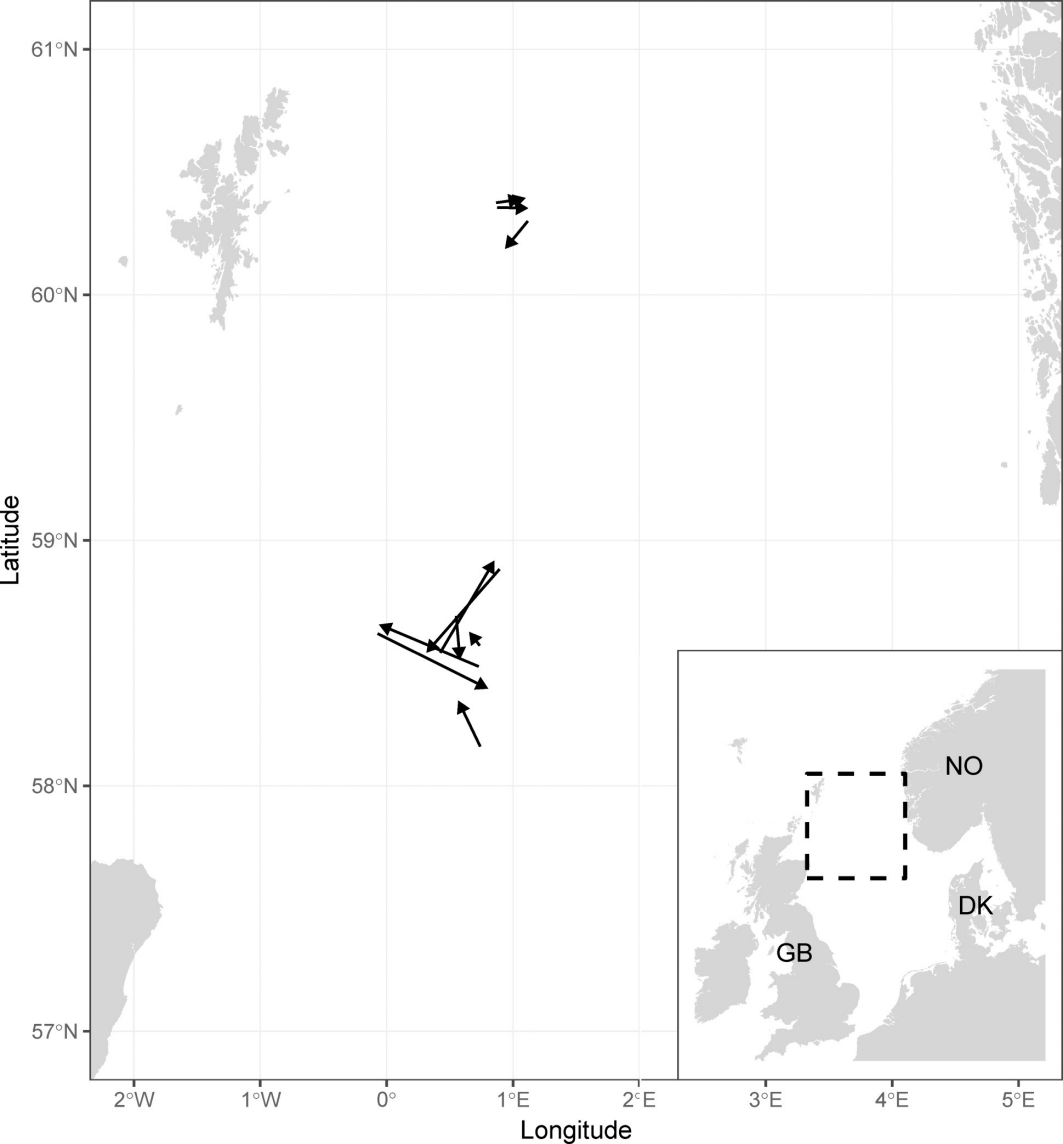
Spatial distribution of the experimental twin-trawl hauls. The four hauls to the north are at the Eastground area, the seven hauls further south are at Fladen ground. Note that the arrows in the figure only indicate start- and endpoint of the individual tows, which were all very similar in duration (between 7:15 and 10:15 hours), but more linear at Fladen ground.

The experimental fishery was carried out from a 70 m long stern-trawler “S364 Rockall”, which is designed to fish with twin-trawls. The trial took place at two commercial Norway pout fishing grounds in the northern part of the North Sea, *Eastground* and *Fladen* ground (Fig 1).

The experiment was conducted between the 31^st^ of October 2018 and 11^th^ of November 2018, which is peak season for the Norway pout fishery. Two identical Egersund 2000 meshes Expo trawls mounted with 80 m bridles and 40 m sweeps were used. The standard grid (*control*) was mounted in one of the two identical trawls and the Excluder (*treatment*) in the other. The sweeps were connected to two Thyborøn type 22 pelagic doors of 11 m^2^ and a 6.5 ton chain-clump between the two trawls and the trawl gear was towed using Dyneema warps. Towing speed was kept between 2.9 and 3.3 knots. The geometry and performance of the gear was continuously monitored using trawl door spread, roll and pitch and codend catch sensors from Marport as well as a PTS Pentagon winch Control System from Rapp Marine. Two technical experts from Rapp Marine participated in the full cruise and ensured optimal winch control and symmetry of the two trawl geometries. The two trawls were switched once during the cruise, so that they both were fished at both sides (port and starboard).

The total catch per trawl was estimated for each haul by the skipper inspecting the catch-indicators in the Refrigerated-Salt-Water tanks (RSW) and noting the change after having pumped in the catch from the respective codends. For each codend catch a fixed amount of 12 full baskets (approx. 340 kg), spread evenly across the pumping period, were sampled from a conveyer belt. This fixed sample size of 12 baskets corresponded to an average total catch weight sampling fraction of 2.4% (Min = 0.9% and Max = 5.2%) across all 22 codend catches. The full sample was sorted and the total sample weight of each species was recorded. For Norway pout a sub-sample of approx. 3.5 kg (Min = 2.4 kg and Max = 5.0 kg) was taken for further treatment (length measurements). After recording species, weight, and length composition (centimeter below), all samples and sub-samples were raised to total catch numbers by weight factors.

### 2.2. Excluder, grid and experimental set up

**Fig 2:**
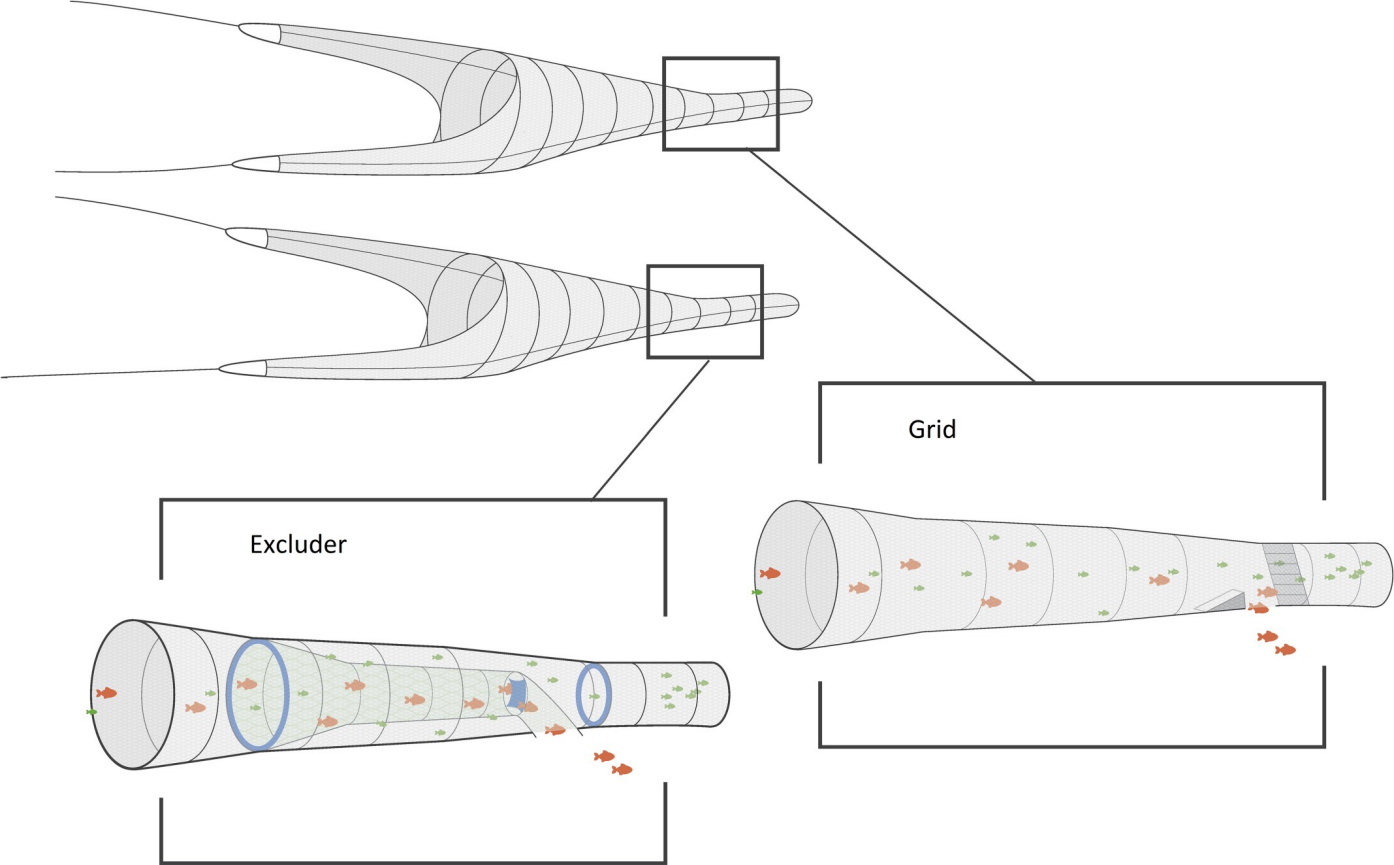
Schematic presentation of the experimental setup. The Excluder was mounted in one of the trawls and the grid in the other trawl of the twin rig. Both systems sort out the larger (red) fish and retain the smaller (green), but based on different selection mechanisms. (Note that the drawing is not true to scale).

The Excluder is an all net section with two PVC kites, inserted as an extension piece of the trawl, which consists of a 30 meter outer-net part and an 11 meter inner selection tube. The inner tube is essentially cone-shaped with an outlet in a bottom panel of the 30 m extension piece, just before the codend. To reach the codend, organisms must pass through the meshes of the inner selection tube and continue along to the codend (Fig 2). The outer-net of the extension piece was made in diamond meshes with a mesh opening of 31.2 mm (SD = 0.7, N = 52). The inner selection tube was made from knotless netting (square meshes) with a mesh opening of 69.9 mm (SD ±1.1 mm, N = 53). The two experimental codends were identical with lengths of 52.8 meters made of 22 mm nominal diamond full meshes in nylon, and a circumference of 1000 meshes. The measured mesh opening of the excluder and the grid codend was 16.9 mm (SD = 0.8, N = 200) and 17.6 mm (SD = 0.9, N = 201), respectively.

A nominal mesh size of 72 mm for the inner selection tube was chosen, based on a fall-through experiment [16] with a stratified length-range of Norway pout, whiting, herring and haddock (approx. 100 individuals of each). A mesh size of 72 mm should ensure that all sizes of the target species can penetrate the square meshes of the inner tube, and end up in the codend, while at the same time excluding as many sizes of the bycatch species as possible. The entrance and exit diameter of the outer-net part of the Excluder was kept fixed at 3.25 m by two cylindrical kites made from heavy PVC cloths. At the end of the 11 m inner tapered selection tube a quadratic PVC sail was mounted across the tube with the purpose of introducing a partial blocking of water current in the surroundings, forcing the fish to either actively bypass the sail or move through the meshes of the inner selection tube.

The Excluder was tested against a 1.6 x 3.6 m grid (six individual sections of 1.6 x 0.6 m lashed together) with 35.5 mm (SD±0.2 mm, N = 55) bar spacing, and made out of plastic. The bar width in the grid was 15.3 mm (SD±0.2 mm, N = 62). The grid was mounted in a 40 mm (nominal full mesh size) extension piece of the second trawl in the twin rig, at an angle of 45° from horizontal with the bottom pointing backwards from the trawl mouth (Fig 2). A guiding panel in 20 mm mesh was inserted in front of the grid leading all fish to encounter the grid at its upper sections, away from the outlet at the bottom of the grid.

### 2.3. Modelling size-dependent catch efficiency

We used the statistical analysis software SELNET [17, 18] to analyze the catch data and conduct length-dependent catch comparison and catch ratio analyses. Specifically, to assess the effect of changing from the standard grid trawl to the Excluder trawl, we used the method from [19] and compared the catch data for the two trawls. This method models the length-dependent catch comparison rate (*CC*_*l*_) summed over hauls:

$${CC}_{l}=\frac{\sum_{j=1}^{m}\left\{\frac{{nt}_{lj}}{{qt}_{j}}\right\}}{\sum_{j=1}^{m}\left\{\frac{{nt}_{lj}}{{qt}_{j}}+\frac{{nc}_{lj}}{{qc}_{j}}\right\}}$$ (1)

where *nc*_*lj*_ and *nt*_*lj*_ are the numbers of fish length measured in each length class *l* for the standard (*control*) and excluder (*treatment*) trawls in haul *j*. *qc*_*j*_ and *qt*_*j*_ are sampling factors quantifying the fraction, based on weight, of the catch in the codends being length-measured in the respective hauls. *m* is the number of hauls where sufficient numbers of each species were caught to be included in the analysis. The functional form for the catch comparison rate *CC(l*,*v)* (the experimental being expressed by Eq (1)) was obtained using maximum likelihood estimation by minimizing the following expression:

$$-\sum_{l}\left\{\sum_{j=1}^{m}\left\{\frac{{nt}_{lj}}{{qt}_{j}}\times ln\left(CC(l,v)\right)+\frac{{nc}_{lj}}{{qc}_{j}}\times ln\left(1.0-CC(l,v)\right)\right\}\right\}$$ (2)

where *v* represents the parameters describing the catch comparison curve defined by *CC(l*,*v)*. The outer summation in the equation is the summation over length classes *l*. When the catch efficiency of the standard and excluder trawl is similar, the expected value for the summed catch comparison rate would be 0.5. Therefore, this baseline can be applied to judge whether or not there is a difference in catch efficiency between the two trawls. The experimental *CC*_*l*_ was modelled by the function *CC(l*,*v)* using the following equation:

$$CC(l,v)=\frac{exp(f(l,v_{0},\ldots ,v_{k}))}{1+exp(f(l,v_{0},\ldots ,v_{k}))}$$ (3)

where *f* is a polynomial of order *k* with coefficients *v*_*0*_ to *v*_*k*_. The values of the parameters *v* describing *CC(l*,*v)* were estimated by minimizing Eq (2), which was equivalent to maximizing the likelihood of the observed catch data. We considered *f* of up to an order of 4 with parameters *v*_*0*_, *v*_*1*_, *v*_*2*_, *v*_*3*_, and *v*_*4*_. Leaving out one or more of the parameters *v*_*0*_*…v*_*4*_ led to 31 additional models also considered as potential models for the catch comparison *CC(l*,*v)*. Among these models, estimations of the catch comparison rate were made using multi-model inference to obtain a combined model [19, 20].

The ability of the combined model to describe the experimental data was evaluated based on the *p*-value. The *p*-value, which was calculated based on the model deviance and the degrees of freedom, should not be < 0.05 for the combined model to describe the experimental data sufficiently well, except for cases for which the data are subject to over-dispersion [19, 21]. Based on the estimated catch comparison function *CC(l*,*v)* we obtained the relative catch efficiency (also named catch ratio) *CR(l*,*v)* between the two trawls using the following relationship:

$$CR(l,v)=\frac{CC(l,v)}{\left(1-CC(l,v)\right)}$$ (4)

The catch ratio is a value that represents the relationship between catch efficiency of the excluder and standard trawl. Thus, if hypothetically the catch efficiency of both trawls is equal, *CR(l*,*v)* should always be 1.0. A hypothetical *CR(l*,*v)* of 1.5 would mean that the excluder trawl is catching 50% more of the species with length *l* than the standard trawl. In contrast, a hypothetical *CR(l*,*v)* of 0.8 would mean that the excluder trawl is only catching 80% of the species with length *l* that the standard trawl is catching.

The confidence limits for the catch comparison curve and catch ratio curve were estimated using a double bootstrapping method [19]. This bootstrapping method accounts for between-haul variability (the uncertainty in the estimation resulting from between haul variation of catch efficiency in the trawls) as well as within-haul variability (uncertainty about the size structure of the catch for the individual hauls including the effect of subsampling). However, contrary to the double bootstrapping method [19], the outer bootstrapping loop in the current study accounting for the between haul variation was performed paired for the excluder and standard trawl, taking full advantage of the experimental design with the trawls being fished in parallel. By multi-model inference in each bootstrap iteration, the method also accounted for the uncertainty due to uncertainty in model selection. We performed 1000 bootstrap repetitions and calculated the Efron 95% [22] confidence limits. To identify sizes of species with significant differences in catch efficiency, we checked for length classes in which the 95% confidence limits for the catch ratio curve did not include 1.0.

Finally, a length-integrated average value for the catch ratio was estimated directly from the experimental catch data using the following equation:

$${CR}_{average}=\frac{\sum_{l}\sum_{j=1}^{m}\left\{\frac{{nt}_{lj}}{{qt}_{j}}\right\}}{\sum_{l}\sum_{j=1}^{m}\left\{\frac{{nc}_{lj}}{{qc}_{j}}\right\}}$$ (5)

where the outer summation covers the length classes in the catch during the experimental fishing period.

## 3. Results

### 3.1 Catch data

A total of 11 hauls were carried out, four with the Excluder on the port side and seven with the Excluder on the starboard side. Haul duration was on average 9 hours and 15 minutes, towing speed approx. 3 knots, and depth approx. 150 m (Table 1). The door spread was approx. 250 meters and total catch (both codends combined) varied between 15 and 70 tonnes.

**Table 1:** Experimental fishery.

Haul no	Area	Duration (hours)	Average speed (knots)	Average depth (m)	Excluder position	Catch in Excluder (kg)	Catch in grid (kg)
1	East ground	07:15	2.9	150	P	8000	8000
2	East ground	08:20	3.0	154	P	35000	35000
3	East ground	08:40	3.2	160	P	10000	10000
4	East ground	09:30	3.1	154	P	15000	15000
5	Fladen ground	09:30	3.3	140	S	17000	35000
6	Fladen ground	09:20	3.2	140	S	9000	8000
7	Fladen ground	09:35	3.3	147	S	18000	22000
8	Fladen ground	09:50	3.1	NA	S	24000	26000
9	Fladen ground	10:00	3.2	142	S	25000	35000
10	Fladen ground	10:15	2.9	145	S	18000	22000
11	Fladen ground	09:40	2.9	144	S	7000	8000

Summary of experimental hauls (P is port side trawl and S starboard side).

Besides Norway pout, a sufficient number of hauls contained sufficient catches of Herring, Mackerel (*Scomber scombrus*), Whiting, American plaice (*Hippoglossoides platessoides*), Lesser silver smelt (*Argentina sphyraena*) and Witch flounder (*Glyptocephalus cynoglossus*), to be included in the analysis (Table 2).

**Table 2:** Catch data.

Haul no	Norway pout		Herring		Mackerel		Whiting		American plaice		Lesser silv. smelt		Witch flounder	
	*nt(qt x 1000)*	*nc(qc x 1000)*	*nt(qt x 100)*	*nc(qc x 100)*	*nt(qt x 100)*	*nc(qc x 100)*	*nt(qt x 100)*	*nc(qc x 100)*	*nt(qt x 100)*	*nc(qc x 100)*	*nt(qt x 100)*	*nc(qc x 100)*	*nt(qt x 100)*	*nc(qc x 100)*
1	347(3.4)	328(4.7)	8(5.3)	71(4.0)	0(5.3)	2(4.0)	1(5.3)	4(4.0)	275(5.3)	262(4.0)	5(5.3)	4(4.0)	11(5.3)	17(4.0)
2	337(0.7)	359(3.8)	67(0.9)	274(0.2)	0(0.9)	4(0.9)	4(0.9)	11(0.9)	146(0.9)	13(0.9)	*	*	0(0.9)	2(0.9)
3	349(3.4)	319(3.5)	0(3.3)	25(3.3)	*	*	0(3.3)	5(3.3)	241(3.3)	294(3.3)	1(3.3)	2(3.3)	9(3.3)	46(3.3)
4	470(1.9)	446(2.0)	21(1.6)	270(1.7)	0(1.6)	18(1.7)	5(1.6)	18(1.7)	132(1.6)	73(1.7)	0(1.6)	1(1.7)	0(1.6)	5(1.7)
5	416(2.1)	340(1.7)	352(2.0)	351(0.4)	1(2.0)	3(1.0)	10(2.0)	109(1.0)	22(2.0)	21(1.0)	5(2.0)	7(1.0)	*	*
6	353(4.9)	477(7.3)	24(3.6)	19(4.0)	*	*	0(3.6)	36(4.0)	17(3.6)	245(4.0)	21(3.6)	39(4.0)	0(3.6)	11(4.0)
7	358(1.7)	341(2.0)	123(1.8)	402(1.5)	1(1.8)	32(1.5)	12(1.8)	115(1.5)	45(1.8)	147(1.5)	11(1.8)	15(1.5)	*	*
8	401(1.4)	347(1.5)	138(1.4)	187(1.3)	1(1.4)	2(1.3)	10(1.4)	126(1.3)	8(1.4)	63(1.3)	1(1.4)	2(1.3)	*	*
9	398(1.6)	369(1.3)	144(1.4)	121(1.0)	1(1.4)	8(1.0)	6(1.4)	109(1.0)	11(1.4)	20(1.0)	4(1.4)	3(1.0)	0(1.4)	2(1.0)
10	257(1.9)	307(2.0)	122(1.9)	361(1.5)	0(1.9)	3(1.5)	5(1.9)	183(1.5)	20(1.9)	100(1.5)	12(1.9)	6(1.5)	*	*
11	351(7.0)	325(8.0)	21(4.5)	26(3.9)	1(4.5)	2(3.9)	22(4.5)	181(3.9)	63(4.5)	153(2.3)	62(4.5)	72(3.9)	0(4.5)	15(3.9)
Sum (*m*)	4037	3958	1020	2107	5	73	75	897	980	1391	122	151	20	98
Sum (*r*)	2.1x10^7^	1.6x10^7^	60792	293404	272	5116	3810	59436	44076	63066	3935	5579	482	3187

Catches per haul in terms of number of fish (*n)* length-measured in each codend (*t* is treatment [Excluder] and *c* is control [grid]). Associated sampling factors (*qt* and *qc*) are also shown. Hauls where neither of the two codend samples contained sufficient numbers for sampling are shown by a

*. Sum (*m*) is the total number of fish length-measured and sum (r) is the raised total catch number.

A number of additional species were caught during the experimental fishery, but in too small quantities to allow for a statistical evaluation of the performance of the excluder compared to the grid. These species were cod (*Gadus morhua*), hake (*Merluccius merluccius*), saithe (*Pollachius virens*), blue whiting (*Micromesistius poutassou*), and argentine (*Argentina sphyraena*).

### 3.2. Catch comparisons for Norway pout

**Fig 3:**
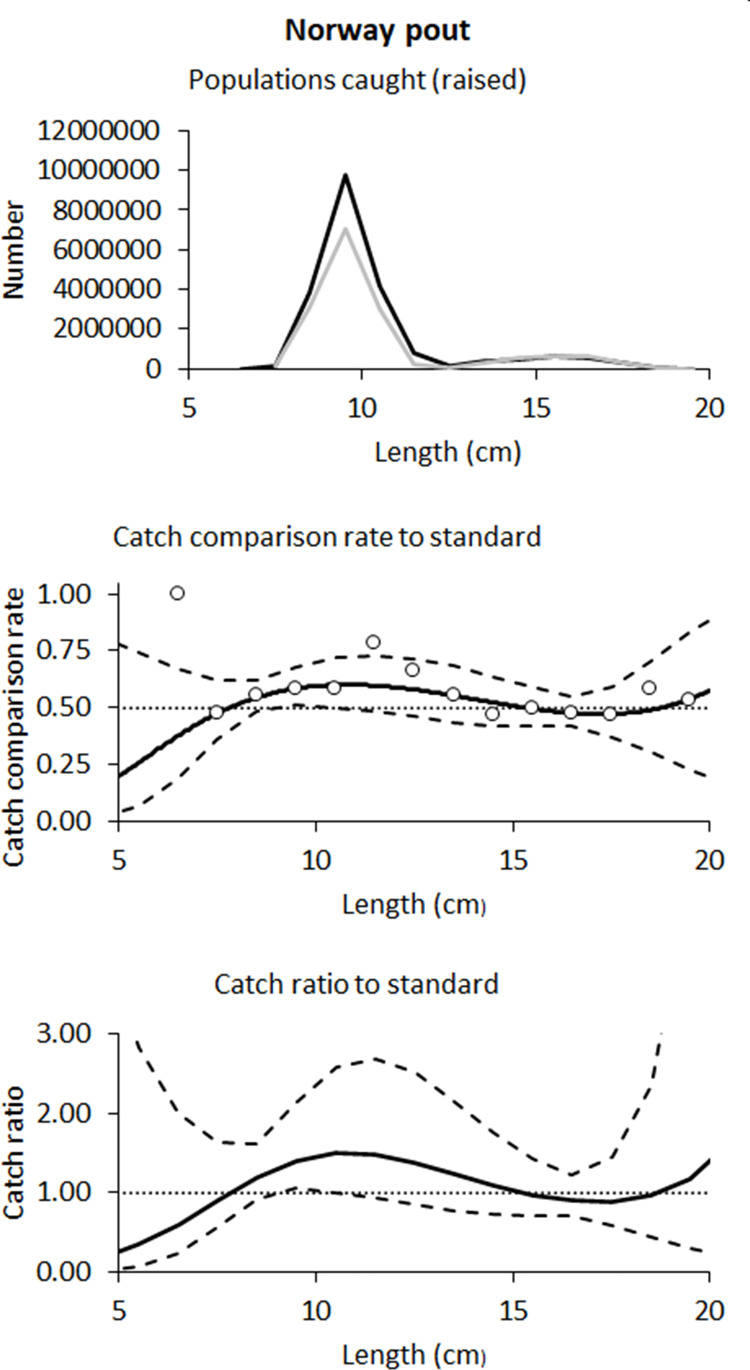
Catch comparisons for Norway pout. The number of Norway Pout caught (top panel), catch comparison rate (middle panel) and catch ratio (bottom panel) for the Excluder compared to the grid. In the top panels the black line shows Excluder trawl catches and the grey line is grid trawl catches. In the lower panels the solid black lines show catch comparison model estimates by length and the dashed lines show the associated 95% confidence intervals.

The estimated catch efficiency for Norway pout was higher in the Excluder for most size classes, however, only at one length class (9.5 cm) was this increase significant (Fig 3). When averaged over sizes, the catch efficiency of the Excluder trawl was estimated to be significantly higher (32% higher) than for the grid trawl (Table 3).

**Table 3:** Catch ratios for Norway pout.

Length (cm)	Catch Ratio:
	Norway pout
7.5	0.90 (0.56–1.64)
8.5	1.19 (0.92–1.62)
9.5	**1.40 (1.05–2.13)**
10.5	1.50 (1.00–2.58)
11.5	1.48 (0.93–2.68)
12.5	1.37 (0.85–2.52)
13.5	1.23 (0.77–2.14)
14.5	1.09 (0.72–1.76)
15.5	0.98 (0.71–1.42)
16.5	0.90 (0.72–1.22)
17.5	0.89 (0.58–1.45)
18.5	0.96 (0.43–2.36)
19.5	1.16 (0.29–5.04)
Average Catch Ratio	**1.32 (1.03–1.95)**
*p*-value	<0.001
Deviance	58.53
DOF	9

Excluder versus grid catch ratio estimates by 1 cm length classes, and averaged over classes, for Norway pout (95% confidence limits in brackets).

### 3.3. Catch comparisons for herring, mackerel and whiting

**Fig 4:**
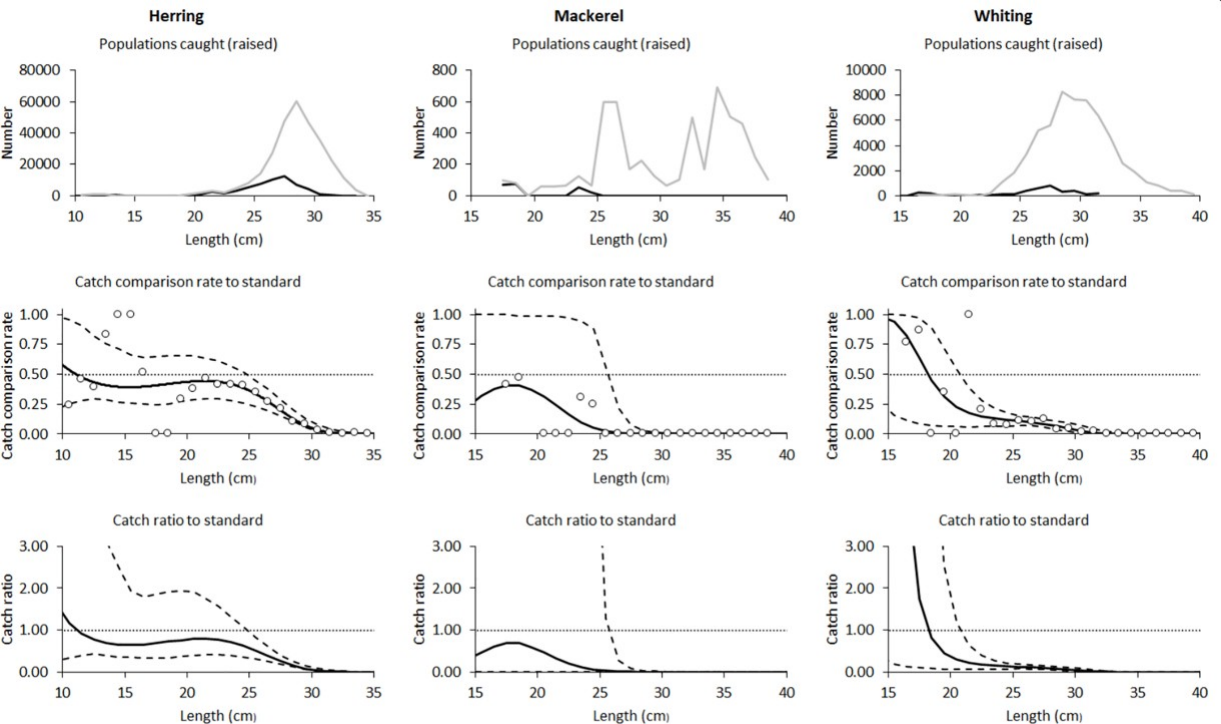
Catch comparisons for herring, mackerel and whiting. The number of Herring, Mackerel and Whiting caught (top panels), catch comparison rate (middle panels) and catch ratio (bottom panels) for the Excluder compared to the grid. In the top panels the black line shows Excluder trawl catches and the grey line is grid trawl catches. In the lower panels the solid black lines show catch comparison model estimates by length and the dashed lines show the associated 95% confidence intervals.

For the three bycatch species herring, mackerel and whiting, there was a large and significant reduction in catches ratios for individuals larger than 25.5, 26.5 and 21.5 cm, respectively, but no significant differences for the smaller individuals (Fig 4). When averaged over length classes, the catch ratios were significantly lower for the excluder trawl for all three species, with the estimated ratios being only 21%, 5% and 6% of the grid trawl catch, for herring, mackerel and whiting, respectively (Table 4).

**Table 4:** Catch ratios for herring, mackerel and whiting.

Length (cm)	Catch Ratio:	Catch Ratio:	Catch Ratio:
	Herring	Mackerel	Whiting
10.5	1.17 (0.34–22.58)	*	*
11.5	0.91 (0.40–9.42)	*	*
12.5	0.77 (0.42–4.50)	*	*
13.5	0.69 (0.40–3.16)	*	*
14.5	0.65 (0.36–2.49)	*	*
15.5	0.65 (0.35–1.94)	*	*
16.5	0.66 (0.33–1.79)	*	4.62 (0.13–208.98)
17.5	0.69 (0.33–1.85)	*	1.75 (0.10–31.58)
18.5	0.72 (0.34–1.92)	0.69 (0.00–92.02)	0.81 (0.08–7.68)
19.5	0.76 (0.37–1.93)	0.60 (0.00–62.64)	0.45 (0.07–2.56)
20.5	0.79 (0.40–1.92)	0.46 (0.00–61.63)	0.29 (0.07–1.17)
21.5	0.80 (0.42–1.75)	0.31 (0.00–81.38)	**0.22 (0.06–0.64)**
22.5	0.78 (0.42–1.56)	0.19 (0.00–40.45)	**0.18 (0.07–0.39)**
23.5	0.72 (0.39–1.32)	0.11 (0.00–17.29)	**0.15 (0.07–0.28)**
24.5	0.63 (0.35–1.06)	0.05 (0.00–7.30)	**0.14 (0.07–0.22)**
25.5	**0.51 (0.30–0.82)**	0.03 (0.00–1.27)	**0.12 (0.08–0.18)**
26.5	**0.37 (0.24–0.59)**	**0.01 (0.00–0.27)**	**0.11 (0.07–0.16)**
27.5	**0.25 (0.18–0.40)**	**0.00 (0.00–0.09)**	**0.09 (0.06–0.14)**
28.5	**0.15 (0.12.0.25)**	**0.00 (0.00–0.03)**	**0.07 (0.04–0.12)**
29.5	**0.08 (0.06–0.15)**	**0.00 (0.00–0.02)**	**0.05 (0.02–0.10)**
30.5	**0.03 (0.02–0.08)**	**0.00 (0.00–0.01)**	**0.03 (0.01–0.08)**
31.5	**0.01 (0.01–0.04)**	**0.00 (0.00–0.00)**	**0.01 (0.00–0.05)**
32.5	**0.00 (0.00–0.02)**	**0.00 (0.00–0.00)**	**0.01 (0.00–0.03)**
33.5	**0.00 (0.00–0.01)**	**0.00 (0.00–0.00)**	**0.00 (0.00–0.01)**
34.5	**0.00 (0.00–0.01)**	**0.00 (0.00–0.00)**	**0.00 (0.00–0.01)**
35.5	**0.00 (0.00–0.00)**	**0.00 (0.00–0.00)**	**0.00 (0.00–0.00)**
36.5	*	**0.00 (0.00–0.00)**	**0.00 (0.00–0.00)**
37.5	*	**0.00 (0.00–0.00)**	**0.00 (0.00–0.00)**
38.5	*	**0.00 (0.00–0.00)**	**0.00 (0.00–0.00)**
Average Catch Ratio	**0.21 (0.11–0.49)**	**0.05 (0.02–0.14)**	**0.06 (0.04–0.11)**
*p*-value	<0.001	<0.001	<0.001
Deviance	72.01	51.65	82.45
DOF	21	17	20

Excluder versus grid catch ratio estimates by 1 cm length classes, and averaged over classes, for herring, mackerel and whiting (95% confidence limits in brackets).

### 3.4. Catch comparisons for American plaice, witch flounder and lesser silver smelt

**Fig 5:**
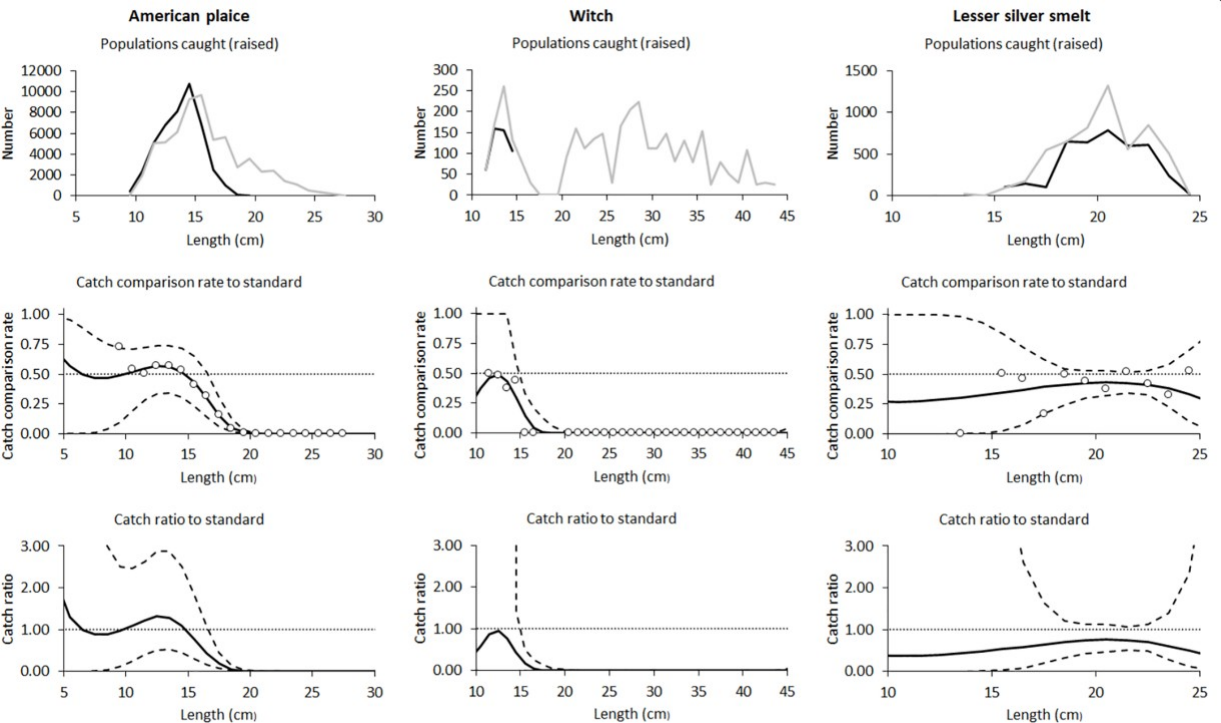
Catch comparisons for American plaice, witch flounder and lesser silver smelt. The number of American plaice, witch flounder and lesser silver smelt caught (top panels), catch comparison rate (middle panels) and catch ratio (bottom panels) for the Excluder compared to the grid. In the top panels the black line shows Excluder trawl catches and the grey line is grid trawl catches. In the lower panels the solid black lines show catch comparison model estimates by length and the dashed lines show the associated 95% confidence intervals.

For the two flatfish bycatch species, American plaice and witch flounder, there was a large and significant reduction in catch ratios for individuals larger than 17.5 and 15.5, respectively, but no significant differences for smaller individuals (Fig 5). When averaged over length classes, catch ratios were significantly lower for the excluder trawl for both species, with the estimated ratios being 71% and 15% of the grid trawl catch, for American plaice and witch flounder, respectively (Table 5). For lesser silver smelt, the estimated catch ratio curve indicated a general reduction in catches when using the excluder trawl, however, not significant for any length-classes.

**Table 5:** Catch ratios for American plaice, witch flounder and lesser silver smelt.

Length (cm)	Catch Ratio:	Catch Ratio:	Catch Ratio:
	American plaice	Witch flounder	Lesser silver smelt
10.5	1.09 (0.24–2.46)	*	*
11.5	1.23 (0.40–2.63)	*	*
12.5	1.31 (0.50–2.87)	*	*
13.5	1.28 (0.53–2.87)	0.76 (0.00–3883.56)	0.43 (0.00–52.36)
14.5	1.09 (0.44–2.51)	0.43 (0.00–1.42)	0.48 (0.01–13.73)
15.5	0.77 (0.31–1.85)	**0.17 (0.00–0.51)**	0.53 (0.03–5.12)
16.5	0.43 (0.17–1.06)	**0.04 (0.00–0.27)**	0.59 (0.08–2.60)
17.5	**0.18 (0.07–0.44)**	**0.01 (0.00–0.14)**	0.65 (0.20–1.64)
18.5	**0.05 (0.02–0.14)**	**0.00 (0.00–0.06)**	0.70 (0.32–1.21)
19.5	**0.01 (0.00–0.03)**	**0.00 (0.00–0.03)**	0.75 (0.43–1.12)
20.5	**0.00 (0.00–0.01)**	**0.00 (0.00–0.01)**	0.76 (0.48–1.12)
21.5	**0.00 (0.00–0.00)**	**0.00 (0.00–0.00)**	0.75 (0.51–1.06)
22.5	**0.00 (0.00–0.00)**	**0.00 (0.00–0.00)**	0.70 (0.48–1.13)
23.5	**0.00 (0.00–0.00)**	**0.00 (0.00–0.00)**	0.61 (0.29–1.39)
24.5	**0.00 (0.00–0.00)**	**0.00 (0.00–0.00)**	0.50 (0.12–2.35)
25.5	**0.00 (0.00–0.00)**	**0.00 (0.00–0.00)**	*
26.5	**0.00 (0.00–0.00)**	**0.00 (0.00–0.00)**	*
27.5	**0.00 (0.00–0.00)**	**0.00 (0.00–0.00)**	*
28.5	**0.00 (0.00–0.00)**	**0.00 (0.00–0.00)**	*
29.5	*	**0.00 (0.00–0.00)**	*
30.5	*	**0.00 (0.00–0.00)**	*
31.5	*	**0.00 (0.00–0.00)**	*
32.5	*	**0.00 (0.00–0.00)**	*
33.5	*	**0.00 (0.00–0.00)**	*
34.5	*	**0.00 (0.00–0.00)**	*
35.5	*	**0.00 (0.00–0.00)**	*
36.5	*	**0.00 (0.00–0.00)**	*
37.5	*	**0.00 (0.00–0.00)**	*
38.5	*	**0.00 (0.00–0.00)**	*
Average Catch Ratio	0.70 (0.27–1.42)	**0.15 (0.00–0.27)**	**0.71 (0.54–0.95)**
*p*-value	0.261	0.983	0.017
Deviance	16.92	12.43	15.49
DOF	14	25	6

Excluder versus grid catch ratio estimates by 1 cm length classes, and averaged over classes, for American place, witch flounder and lesser silver smelt (95% confidence limits in brackets).

## 4. Discussion

For all bycatch species analyzed it was estimated that the Excluder significantly reduced unwanted catches, either for specific fish lengths or averaged across all length-classes, when compared to the grid. The average catch ratio of the target species, Norway pout, was 32% (CI: 3–95%) higher in the Excluder trawl.

### 4.1. Selectivity of the two systems

From related sorting grid and square mesh selectivity studies (e.g. [23, 24]) and the fall-through experiment of this study, it was hypothesized that the length-based rejection rate of the 70mm square meshes of the Excluder would be substantially higher (round fish L50 of approx. 25 cm) than for the 35mm bar distance of the grid (round fish L50 of approx. 35 cm). This hypothesis was corroborated in the experimental fishery, where the Excluder had substantially lower catches of all six bycatch species compared to the grid. The reduction in total catch numbers across all length-classes ranged from 29% to 95%. Five bycatch species exhibited significant length-dependent reductions, where only larger individuals (above approx. 25 cm for round fish and approx. 17 cm for flatfish) were sorted out more in the Excluder than by the grid. These substantially higher bycatch reductions in the Excluder confirm the hypothesis of a difference in the length-dependent rejection rate of the two sorting systems.

While it was anticipated that the Excluder would reduce bycatch compared to the grid, the effect on target catch was less predictable. With an estimated 32% higher catch efficiency in the Excluder trawl, the experimental data provided strong support to the overarching design principle and hypotheses behind the Excluder system; that the large contact area available for the selection process enables a more efficient separation of target species from bycatch species than in grid-based sorting systems with smaller contact areas. In previous experiments with rigid sorting grids for separating target and bycatch species, underwater video footage has demonstrated general problems with fish-clogging and associated loss of grid function [25, 26]. The surface area of the Excluder inner tube is roughly 15 times larger than the 5.8 m^2^ surface area of the experimental grid, which is likely to significantly reduce the risk of clogging and related loss of function, and hence improve the catch efficiency towards the smaller-sized target species fish. In this comparison of contact area it is should be considered that that the contact rate is likely to vary along the length of the Excluder.

### 4.2. Methodological improvements

Ideally the Excluder and grid trawls would have been deployed approx. the same number of times on each side (port and starboard) on each of the two fishing grounds visited, but due to the size of the gear, switching trawls was a very time consuming task and therefore only possible to perform once, while steaming from the first fishing ground to the second. The experimental fishery being conducted as a commercial trip (i.e. that the trial costs were largely to be covered by the catches) was a contributing factor to the low number of trawl switches. A downside of not being able to balance the port and starboard positions of the experimental gear is an increased risk of confounding effects in the analysis. The summary of the experimental hauls (Table 4) might give the impression that trawl position (port/starboard) rather than sorting system (Excluder/grid) explains the difference in total catches, but two aspects contradict this: i) the geometry of the twin trawl gear was continuously monitored and maintained by experts using state-of-the-art symmetry sensors, ii) a significant increase in whiting abundance (and a resulting lower total catch in the Excluder trawl compared to the grid trawl) that coincided exactly with the port/starboard shift of the experimental gears (Table 2).

Another characteristic of the Norway pout fishery, which complicated the experimental data collection, is that the very large gear and catch size does not allow weighing of all the catch. Instead each codend catch (Excluder and grid) was estimated to the nearest 1000 kg by the skipper based on the catch volume markers on the sides of the RSW-tanks. This procedure is more crude and more subject to uncertainty than exact measurements, but the approach is similar to how total catch weights are estimated and reported (legally binding) in the official logbooks, which adds confidence to the methodology and decreases the likelihood of any unintended bias.

The two codends of the experimental fishery were stored immediately after the sea trials, and when later measured they did not have similar mesh openings (Excluder codend 12.6 mm [SD = 2.2] and grid codend 16.0 mm [SD = 1.5], N = 50). This was somewhat surprising as both codends were made of identical 22 mm nominal diamond meshes in nylon, and different treatments of the two codends (the Excluder codend unpacked and exposed to the sun for ½ a day before measurements and the grid codend measured in wet condition directly from the storage box) were suspected to have biased the measurements [27]. Consequently, the two codends were revisited and measured again after ensuring identical pre-treatment of both codends (soaking and stretching), which resulted in similar mesh openings of 16.9 mm (SD = 0.8, N = 200) for the Excluder codend and 17.6 mm (SD = 0.9, N = 201) for the grid codend.

### 4.3. Excluder handling and safety

Opposite to the grid, the Excluder has the advantage of being made of flexible material, enabling it to be reeled directly on the net drum together with the trawl. This ease of handling of the Excluder is particularly advantageous in the case of twin-trawling–as experienced by the commercial and scientific crew in the experimental fishery of this study–where the handling of two rigid grids, one in each trawl, would provide a challenge, both in terms of operation safety and handling time [14]. Furthermore, if implemented, the ease of handling of the Excluder would likely also increase compliance in the fishery, as compatibility between regulations and traditional fishing practices (as well as the efficacy of imposed regulations) has been shown to facilitate acceptance [28].

### 4.4. Implications for the Norway pout fishery

The main objective of the study was to develop and test an easy handled, high-safety, netting-based sorting system, capable of minimizing unintended bycatch while efficiently retaining the target species in the Norway pout fishery. With the Excluder system and the results of the sea trial this aim has been achieved, and the technological basis of a more selective Norway pout fishery without rigid and inhibitory sorting grids is in place. Furthermore this project aim was achieved in a multidisciplinary approach involving academic expertise, gear manufacturers and the commercial fishery, which enhances the sense of industry buy-in and the likelihood of uptake in the fishing fleet (since the completion of the field trial the participating netmaker, Tor-Mo Trawl ApS, has received orders for two Excluders for the Norway pout fishery).

Assuming that the species and size distributions encountered during the experimental fishing are representative of those in the commercial fishery, a widespread replacement of the grid with the Excluder would not only lead to a substantial reduction in bycatches, but also an increase in target species CPUE. Bycatch issues and low catch rates were initially identified as key barriers for an improved quota capitalization in this fishery and therefore implementation of the Excluder would likely increase the landings of this currently under-utilized resource, as well as significantly improve the sustainability of the fishery.

### 4.5. Perspectives

The Excluder used in this Norway pout experiment was a first design and therefore further improvement to increase its efficiency, both in terms of bycatch reduction and target species efficiency, could be possible. In the present setup the inner selection tube was 11 m but could potentially be extended in future designs, so that the sorting area would be increased even further. The selective properties of the Excluder could likely also be improved by optimizing the mesh size of the inner section. In this experiment, we tested a 70 mm mesh opening, which was assessed to represent a sensible tradeoff between reduction of unwanted bycatch and loss of target species. By either increasing or decreasing the mesh opening the current balance can be shifted to either side, potentially providing a better tradeoff result. The tested Excluder was designed specifically for the small-meshed Norway pout fishery in the North Sea area with the objective of retaining this small-sized target species and sorting out larger ones, but it also represents a potential solution to similar sorting issues, grid-handling and clogging problems in other trawl fisheries. In the North east Atlantic other obvious sorting issues to explore are the unwanted saithe (*Pollachius virens*) bycatches in fisheries targeting herring and the bycatches of larger pelagic species, such as mackerel, in the fisheries targeting sandeel (*Ammodytes marinus*), and globally a substantial number of additional trawl fisheries with similar sorting issues would likely benefit from replacing grid-based systems with tailored netting-based Excluder systems.

## Supporting information

S1 Catch Data for individual hauls. The catch data consist of count data for number of individuals of all species caught with respectively the Excluder codend and the Grid codend, for each size class (length).
(XLSX)
